# Dose–effect relationship of stereotactic body radiotherapy in non-small cell lung cancer patients

**DOI:** 10.1186/s13014-022-02183-3

**Published:** 2022-12-23

**Authors:** Fei Li, Hairong Jiang, Mingwei Bu, Xin Mu, Hongfu Zhao

**Affiliations:** 1 Department of Radiation Oncology, China-Japan Union Hospital of Jilin University, No. 126, Xiantai Street, Changchun, 130033 Jilin People’s Republic of China; 2 Department of Geriatrics, Jilin City Hospital of Chemical Industry, Jilin, 130022 Jilin People’s Republic of China; 3 Department of Radiation Oncology, Guowen Medical Corporation Changchun Hospital, Changchun, 130028 Jilin People’s Republic of China; 4 Department of Radiation Oncology, Jilin City Hospital of Chemical Industry, Jilin, 130022 Jilin People’s Republic of China

**Keywords:** Non-small cell lung cancer, Stereotactic body radiotherapy, Dose–effect relationship

## Abstract

**Objective:**

To establish the dose effect relationship between the dose parameters of stereotactic body radiation therapy (SBRT) for early non-small cell lung cancer (NSCLC) and the local tumor control rate.

**Materials and methods:**

A comprehensive literature search was conducted using PubMed, the Web of Science and the Cochrane databases to determine the articles treated with SBRT in early-stage NSCLC. Original studies with complete prescription dose information, tumor local control rate and other important parameters were screened and reported. Probit model in XLSTAT 2016 was used for regression analysis, and *P* < 0.05 was set as a statistically significant level.

**Results:**

After literature screening, 22 eligible studies were included in probit model regression analysis, involving 1861 patients. There is no significant dose effect relationship between nominal BED_10_ and peripheral BED_10_ versus 3 years local control probability. There were significant dose effect relationships between the center BED_10_ and the average BED_10_ versus the 3 years local control probability, with *P* values are 0.001 and < 0.0001, respectively. According to the results of this model, the 3 years local control rate of 90.5% (87.5–92.1%) and 89.5% (86.7–91.0%) can be expected at the center BED_10_ of 180 Gy or the average BED_10_ of 140 Gy, prospectively.

**Conclusions:**

For NSCLC treated with SBRT, more attention should be paid to the central dose and average dose of PTV. A set of clear definition in the dose prescription should be established to ensure the effectiveness and comparability of treatment.

**Supplementary Information:**

The online version contains supplementary material available at 10.1186/s13014-022-02183-3.

## Introduction

Stereotactic body radiotherapy (SBRT) is the standard treatment for inoperable early non-small cell lung cancer (NSCLC), with good local control rate and low incidence of side effects [1, 2]. SBRT has also achieved encouraging outcomes in patients with operable early NSCLC [3, 4]. The total physical dose varies greatly due to different fractionation [5, 6]. Even if the fractionation and the nominal prescription dose are the same, there is significant difference for near maximum dose or the average dose in the planning target volume due to the absence of the uniform standard for the selection of prescription dose isodose line [7]. In SBRT of early stage NSCLC, higher maximum dose in target volume can not only improve local control rate, but also reduce cancer specific death and promote overall survival (OS), without increasing toxicity [8]. Several studies have attempted to determine the most relevant dose parameters for tumor local control in SBRT of NSCLC, and tried to recommend the prescription dose [5, 6]. However, it is regrettable that the optimal dose prescription is still unclear.

In this study, published articles on SBRT in NSCLC were comprehensively searched, and the dose effect relationship between prescription dose and tumor local control rate was established through Probit model.

## Materials and methods

### Data sources and search strategy

A comprehensive literature search was conducted using the PubMed, the Web of Science and the Cochrane databases to determine the published articles of NSCLC patients treated with SBRT. We searched “stereotactic ablative radiotherapy”, “stereotactic body radiotherapy” “non-small cell lung cancer” or their synonym in the title or abstract, and the search was restricted to English-language (Additional File 1: Table S1). Our last literature search was on September 30, 2022.

### The inclusion criteria

1. The enrolled patients were early-stage (T_1-2_ N_0_) NSCLC.
2. The treatment conforms to the SBRT technical specifications in the AAPM TG101 report.
3. The original study of complete prescription dose information and tumor local control rate was reported.
4. The median follow-up time should be at least 24 months and the sample size of patients should be at least 20.

### The exclusion criteria

1. Review articles, articles about recommendations, consensus or guidelines.
2. Irrelevant articles or articles focusing on the research of radio diagnostics, medical physics and radiobiology.
3. The study included more than one dose fractionation, and did not report tumor local control for a cohort of single dose fractionation.
4. Proton beam stereotactic radiotherapy.
5. Articles focused on the combination of SBRT and other treatments, such as surgery, or the use of SBRT only as boost after conventional radiotherapy, or a salvage treatment for patients of recurrence.
6. Non-English articles.

### Data extraction

After removing the duplicate literature, we screened the literature by title and abstract, and the remaining literature was screened by full text. When the patient data reported in the literature overlap, we selected the latest and most complete data. Literature screening and data extraction are conducted independently by two authors according to the inclusion and exclusion criteria, and objections are resolved through negotiation. The following data were extracted from all selected studies: 1. Article information: first author, publication year, country, patient treatment time and patient number; 2. Patient and tumor characteristics: median age, tumor stage, medically operable or inoperable, internal target volume (ITV) delineation and planning target volume (PTV) margin; 3. Treatment characteristics: prescription (nominal) dose, peripheral (near minimum) dose, central (near maximum) dose, number of minutes, interval time between fractions; 4. Three years local control rate. If 3 years local control rate is not available in the literature, obtained from Kaplan Meier curve is also recognized. If multiple prescription doses and corresponding local control are introduced in the article, subgroups data will be included in the analysis. The biologically effective doses (BED) were calculated using the linear quadratic (LQ) equation: BED_10_ = nd[1 + d/(α/β)], where d represents the fraction dose, n represents the fractions, and α/β equals to 10. Due to the high heterogeneity of the PTV dose in SBRT, in order to facilitate the dose effect analysis, this study defines several doses: nominal dose, which is the prescription dose; central dose, which is the maximum dose of PTV, isocenter dose or the minimum dose to the 2% volume of the maximally exposed region in PTV (D2); peripheral dose is the minimum dose of PTV or D95–98. When central dose BED_10_ and peripheral dose BED_10_ data are available, the average value is defined as average dose BED_10_.

### Regression analysis

Probit model regression analysis was conducted by XLSTAT 2016 (Addinsoft, Paris, France). Select the mean or median dose BED_10_ as the quantitative dose. The number of patients is considered as observation weights in the dose effect analysis panel. The statistical significance was set at the level of *P* < 0.05. Subgroup analysis was conducted according to the country, type of study design, treatment era, operable or inoperable, proportion of male patients, median age, patient immobilization mode, CT scanning mode, PTV margin, number of fractions, interval time between fractions, treatment equipment, tissue heterogeneous correction or not, follow-up time.

## Results

### Description of the included studies

After comprehensive search, no article about regression analysis on dose effect relationship between dose and tumor local control rate based on published data was found. A total of 3699 potential related studies were identified using a systematic literature retrieval strategy. After duplicates removed, 22 eligible studies were obtained through title, abstract and full text screening and included in probit model regression analysis, involving 1861 patients in total, as shown in Additional File 1: Fig. S1. These included studies were from 6 countries, with the most published studies coming from the United States (nine), followed by Japan (seven), Italy and Sweden (two each), Spain and Canada (one each). Among them, there are 13 prospective studies, 9 retrospective studies. The main characteristics of the included studies are presented in Table 1.

**Table 1:** The main characteristics of the included studies

First author (year)	Country	Study design	Treatment time	N	Male/female	Operable	Age	Immobilization and breath control	CTV/ITV delineation	PTV margin (mm)	Nominal/central/peripheral dose (Gy)	Fractions	Interval time	Treatment equipment	THC	Nominal/central/peripheral/average BED (Gy)	Median follow-up (m)	3-years local control (%)
Baumann (2009) [9]	Sweden	PS	2003–2005	57	31/26	No	75	SBF, AC	CTV = GTV + (1–2)	5–10 in TP; 10 in LP	45/66/45	3	2 days	LA, 6MV, 5–9 NCB or CB	Yes	112.5/211.2/112.5/161.9	36	93
Fakiris (2009) [10]	U. S	PS	2003–2008	34 36	NR	No	NR	SBF	CTV = GTV	TP: 5; LP: 10	60/75/60 66/82.5/66	3	NR	LA, 10–12 NCB	No	180/262.5/180/221.3 211.2/309.4/211.2/260.3	50.2	88.1 88.1
Takeda (2009) [11]	Japan	RS	2001–2007	63	40/23	Both	78	NR	Long-scan-time CT	6–8	50/62.5/50	5	Total 5–7 days	LA, 10 arc dynamic conformal	Yes	100/140.6/100/120.3	31	93.7
Baba (2010) [12]	Japan	PS	2004–2008	85 37	54/31 29/8	Both	77 78	Body Fix	Normal breathing, expiratory holding and inspiratory holding CTs, CTV = GTV	TP: 5; LP: 10	48/48/38.4 52/52/41.6	4 4	Twice per week	LA, 3 CB and 4 NCB	Yes	105.6/105.6/75.3/90.4 119.6/119.6/84.9/102.2	26	81 74
Ricardi (2010) [13]	Italy	PS	2003–2007	62	52/10	No	73.7	SBF	Long-scan-time CT, CTV = GTV	TP: 5; LP: 10	45/56.25/45	3	2 days	LA, 6–8 NCB	Yes	112.5/161.7/112.5/137.1	28	87.8
Timmerman (2010) [14]	U. S	PS	2004–2006	55	21/34	No	72	NR	4D-CT, CTV = GTV	TP: 5; LP: 10	54/67.5/54	3	Total 1.5–2 weeks	LA, 4–10 MV	No	151.2/219.4/151.2/185.3	34.4	97.6
Shirata (2012) [15]	Japan	RS	2005–2009	45	NR	NR	77	Vacuum pillow	Long-scan-time CT, ITV = GTV + 0 ~ 5	PTV = ITV + 5–10	48/48/43.2	4	4–6 days	LA, NCB dynamic arcs	Yes	105.6/105.6/89.9/97.7	30.4	100
Rosen (2014) [16]	U. S	PS	2005–2010	20 59	33/46	No	73	Body FIX, AC	4D-CT, PET-CT fusion for GTV, 6–10 mm margin	6	48/48/48 60/6060	5	1 day	Tomotherapy	NR	105.6/105.6/105.6/105.6 132/132/132/132	27	85.0 96.6
Lindberg (2015) [17]	Sweden	PS	2003–2005	57	26/31	No	75.2	SBF, AC	CTV = GTV + 1 ~ 2	TP: 5; LP: 10	45/67.2/45	3	NR	LA, 6MV	NR	112.5/217.7/112.5/165.1	41.5	91.8
Nagata (2015) [18]	Japan	PS	2004–2008	164	120/44	Both	78	NR	CTV = GTV	5	48/48/42	4	Total 4–8 days	LA, NCB or dynamic arc	Yes	105.6/105.6/86.1/95.9	47/67**	86.6
Navarro-Martin (2016) [19]	Spain	PS	2008–2012	38	36/2*	No	74	Thermoplastic masks	4D-CT	6	54/70.2/54	3	8–14	LA, 7 NCB or arcs	Yes	151.2/234.4/151.2/192.8	36	94
Shaverdian (2016) [20]	U. S	RS	2009–2013	110	NR	NR	75	Vacuum pillow	4D-CT, MIP	TP: 3; LP: 6	54/65.6/54	3	2 days	LA, CB and NCB	Yes	151.2/209.0/151.2/180.1	28.9	100
Tsurugai (2016) [21]	Japan	RS	2005–2014	155	99/56	Both	77	SBF, AC	Long-scan-time CT	5	48/48/42.2	4	NR	LA, 6–7 NCB	Yes	105.6/105.6/86.7/96.2	34.7	90.5
Sun (2017) [22]	U. S	PS	2005–2013	65	32/33	Both	71.8	Deep inspiration breath hold	4D-CT, MIP	NR	50/–/50	4	NR	LA, 6–12 CB or NCB	NR	112.5/–/112.5/–	86	95.4
Cummings (2018) [23]	U. S	PS	2007–2015	65 98	30/35 44/54	NR	76 77	ITV > 0.5 cm: respiratory gating	CTV = GTV, 4D-CT	5	30/–/30 50/50/40	1 5	1 fraction per day	LA, 11 NCB	No	120/120/–/– 100/100/72/86	24 40	84.0 82.8
karasawa (2018) [24]	Japan	RS	2003–2010	56	39/17	No	79	SBF	Long-scan-time CT, margin 5 mm	5	48/48/42	4	Total 1 week	LA, 6MV	Yes	105.6/105.6/86.1/95.9	127	78.2
Ma (2018) [25]	U. S	RS	2007–2015	94	47/47	NR	76.2	Expiration breath hold or free breathing 4D-CT	CTV = GTV, 4D-CT	5	60/80/60	3	2 days	LA, 11 NCB	NR	180/293.3/180/236.7	29.3	73.5
Raghavan (2018) [26]	U. S	RS	2009–2014	140	25/24	No	70 +	Vacuum pillow	4D-CT, MIP	TP: 3; LP: 6	54/67.5/54	3	2 days	LA, 6MV	No	151.2/219.4/151.2/185.3	38.8	93.4
Bezjak (2019) [27]	Canada	PS	2009–2013	38 33	15/23 13/20	No	71 72	NR	4D-CT	4D-CT: 5; Non 4D-CT: TP: 5; LP: 10	57.5/71.9/57.5 60/75/60	5	2–3 days	LA, 3D-CRT or IMRT	Yes	123.6/175.3/123.6/149.5 132/187.5/132/195.8	37.9	86.7 84.7
Nicosia (2019) [28]	Italy	RS	2010–2017	44	29/15	No	75	NR	4D-CT	4–5	30/31.6/30	1	NR	LA, 7–9 beams	NR	120/131.5/120/125.7	34	87.8
Devpura (2021) [29]	U. S	PS	NR	55	29/26	No	75	Free breathing	4D-CT, CTV = GTV	TP: 3; LP: 6	48/50.5/48	4	2 days	LA	Yes	105.6/114.3/105.6/114.3	24	93
Ryuno (2022) [30]	Japan	RS	2010–2019	96	61/35	Both	77	Vacuum pillow, RTS	4D-CT	2	54/72/54	3	NR	Cyberknife	NR	151.2/244.8/151.2/198	27	95.8

*N* number *CTV* clinical target volume, *ITV* internal target volume, *PTV* planning target volume, *THC* Tissue heterogeneity correction, m months, *PS* prospective study, *SBF* stereotactic body frame, *AC* abdominal compression, *TP* transversal plane, *LP* longitudinal plane, *LA* linear accelerator, *NCB* noncoplanar beam, *CB* coplanar beam, *U. S.* United States, *NR* Not reported, *RS* retrospective study, *MIP* maximum intensity projection, *GTV* gross target volume, *4D-CT* four-dimensional computed, *3D-CRT* three-dimensional conformal radiotherapy, *IMRT* intensity modulated radiotherapy, *RTS* respiratory tracking system

^*^4 patients did not report gender

^**^The inoperable group and the operable group reported the follow-up time respectively

### Probit analyses

**Fig. 1:**
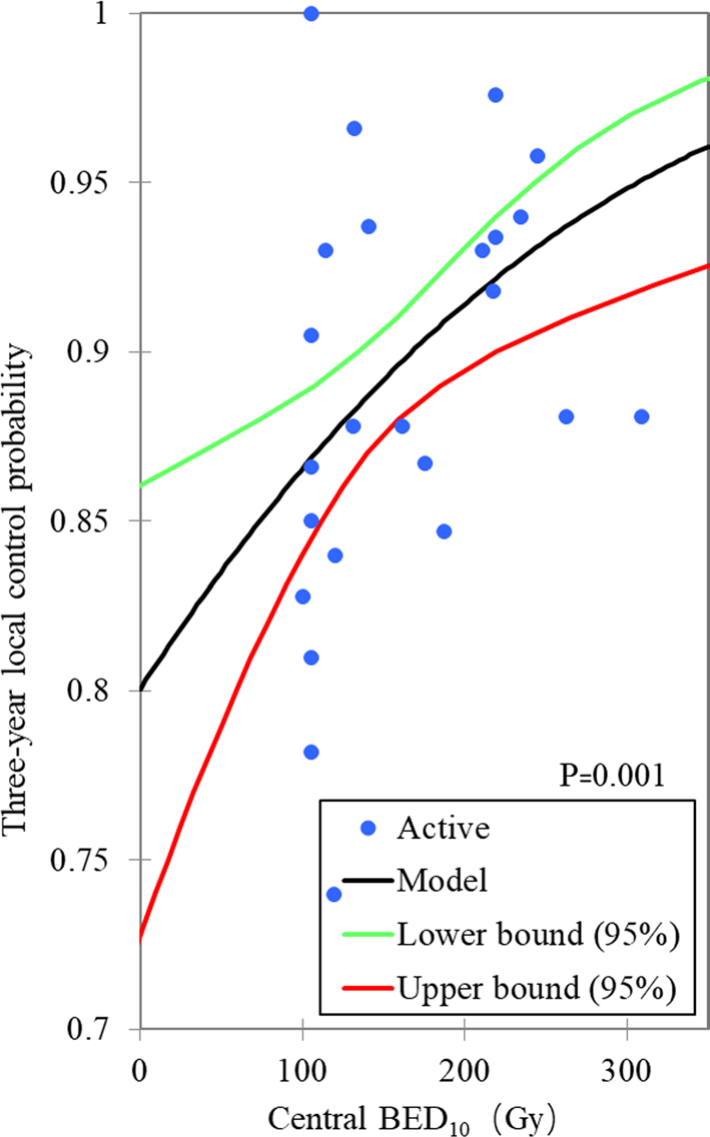
The probit model for the relationship between central BED_10_ and 3-years local control

**Fig. 2:**
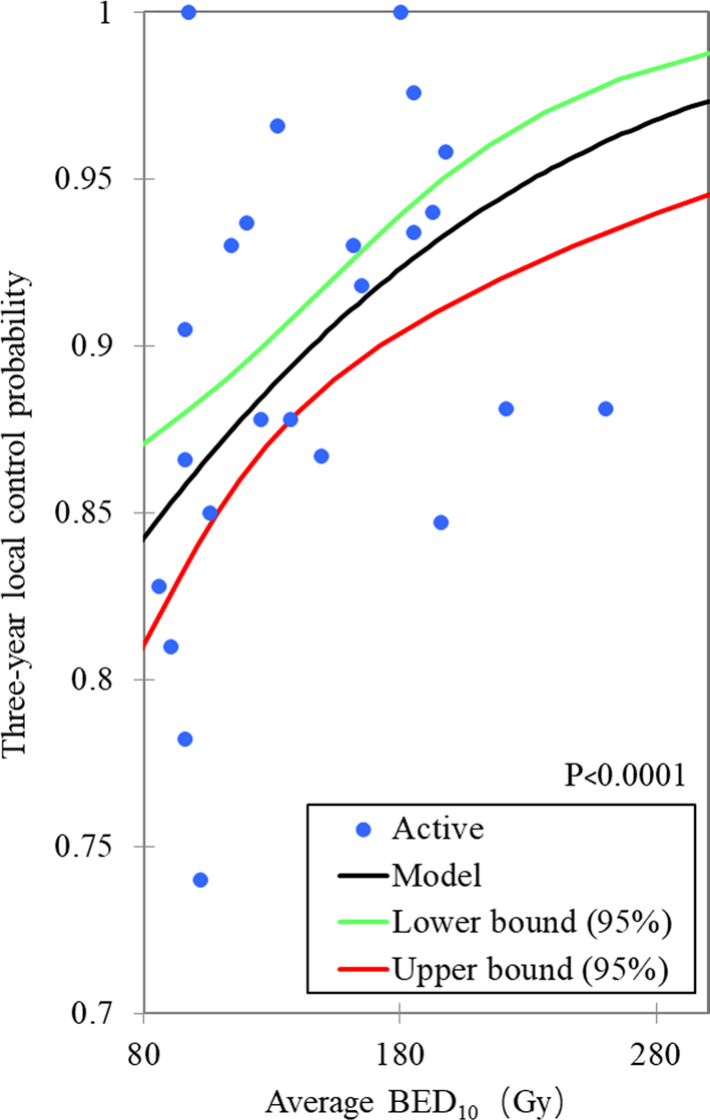
The probit model for the relationship between average BED_10_ and 3-years local control

The range of prescription dose is 30–66 Gy, the fractions range is 1–5, and the actuarial or rough 3-years local control rate is 73.5–100.0%. There is no significant dose effect relationship between nominal BED_10_ and peripheral BED_10_ versus 3-years local control probability. There were significant dose effect relationships between the center BED_10_ and the average BED_10_ versus the 3 years local control probability, with *P* values are 0.001 and < 0.0001, respectively, as shown in Figs. 1 and 2. According to this model, the central BED_10_ and the average BED_10_ corresponding to probabilities of 90% 3 years local control were 167.5 Gy_BED10_ (135.0–219.6 Gy_BED10_) and 145.7 Gy_BED10_ (126.7–172.2 Gy_BED10_) respectively. The 3 years local control rate of 90.5% (87.5–92.1%) and 89.5% (86.7–91.0%) can be expected at the center BED_10_ of 180 Gy and the average BED_10_ of 140 Gy, prospectively. The Probit analyses based on subgroups show that the central BED_10_ and peripheral BED_10_ have most cumulative number of significance (Ten each), followed by the average BED_10_ (Eight), and nominal BED_10_ (Six), as shown in Table 2.

**Table 2:** The Probit analyses based on subgroups between BED_10_ and 3-years local control

Parameter	Nominal dose BED_10_ (Gy_BED10_)			Peripheral dose BED_10_ (Gy_BED10_)			Central dose BED_10_ (Gy_BED10_)			Average dose BED_10_ (Gy_BED10_)		
	s (p)	ED90 (95% CI)	*P*	s (p)	ED90 (95% CI)	*P*	s (p)	ED90 (95% CI)	*P*	s (p)	ED90 (95% CI)	*P*
*Country*												
United States	9 (831)	143.0 (–, –)	0.331	8 (766)	182.1 (–, –)	0.653	8 (766)	166.0 (–, –)	0.318	7 (701)	176.7 (–, –)	0.280
Japan	7 (701)	122.5 (–, –)	0.069	7 (701)	100.2 (89.7, 123.6)	**0.002**	7 (701)	144.0 (117.2, 227.6)	**0.007**	7 (701)	121.8 (103.5, 171.0)	**0.004**
*Study design*												
Prospective	13 (1058)	148.5 (–, –)	0.118	12 (993)	125.1 (104.1, 176.9)	**0.003**	12 (993)	200.0 (157.1, 394.0)	**0.010**	11 (928)	167.0 (132.5, 310.1)	**0.011**
Retrospective	9 (803)	148.0 (–, –)	0.105	9 (803)	153.1 (–, –)	0.301	9 (803)	218.2 (–, –)	0.136	9 (803)	184.6 (–, –)	0.178
*Treatment era*												
Before 2010	9 (695)	185.5 (–, –)	0.665	9 (695)	127.3 (–, –)	0.100	9 (695)	190.1 (–, –)	0.083	9 (695)	157.8 (–, –)	0.084
After 2005	12 (1100)	146.2 (–, –)	0.439	11 (1035)	235.3 (–, –)	0.792	11 (1035)	181.9 (–, –)	0.465	10 (970)	182.3 (–, –)	0.350
*Operable or not*												
Both	6 (665)	118.4 (–, –)	0.064	6 (665)	98.8 (88.2, 116.3)	**0.001**	5 (600)	145.9 (117.5, 227.1)	**0.007**	5 (600)	123.3 (103.5, 174.1)	**0.005**
Inoperable	12 (784)	120.5 (–, –)	0.237	12 (784)	121.2 (–, –)	0.097	12 (784)	165.3 (–, –)	0.125	12 (784)	144.4 (–, –)	0.152
*Proportion of male patients*												
≥ 50%	11 (907)	86.1 (–, –)	0.061	11 (907)	9.2 (–, –)	0.397	11 (907)	–67.9 (–, –)	0.439	11 (907)	–30.7 (–, –)	0.435
< 50%	8 (729)	126.1 (114.2, 144.4)	**0.002**	7 (664)	115.9 (100.6–133.6)	**0.0001**	7 (664)	173.7 (146.2, 219.3)	**0.001**	6 (599)	149.3 (124.0, 186.1)	**0.001**
*Median age*												
> 75	10 (1015)	68.4 (–6622.2, 100.4)	**0.048**	9 (950)	–63.3 (–, –)	0.451	10 (1015)	–221.2 (–, –)	0.478	9 (950)	–116.4 (–, –)	0.441
≤ 75	10 (636)	110.3 (82.9, 120.4)	**0.002**	10 (636)	110.3 (82.9, 120.4)	**0.002**	9 (571)	123.5 (− 452.0, 161.7)	**0.034**	9 (571)	116.0 (–, –)	0.051
*Immobilization*												
SBF	6 (457)	− 294.5 (–, –)	0.945	6 (457)	291.9 (–, –)	0.851	6 (457)	246.5 (–, –)	0.459	6 (457)	221.8 (–, –)	0.580
BodyFix	2 (201)	125.3(115.9, 211.6)	**0.025**	2 (201)	108.0 (94.0, 153.1)	**0.003**	2 (201)	125.3 (115.9, 211.6)	**0.025**	2 (201)	115.1 (104.6, 153.0)	**0.005**
VP	5 (453)	208.2 (–, –)	0.405	5 (453)	204.8 (–, –)	0.316	5 (453)	541.0 (–, –)	0.567	5 (453)	349.2 (–, –)	0.472
AC	4 (348)	105.8 (–, –)	0.096	4 (348)	88.7 (–, –)	0.170	4 (348)	44.7 (–, –)	0.753	4 (348)	82.9 (–, –)	0.396
*CT scan*												
4D-CT	13 (1432)	146.4 (–, –)	0.075	11 (949)	60.9 (–, –)	0.689	12 1367)	216.4 (–, –)	0.130	10 (884)	248.3 (–, –)	0.737
*PTV margin*												
≥ 5 mm	16 (1351)	43.3 (–, –)	0.211	15 (1286)	− 949.9 (–, –)	0.911	16 (1351)	− 250.9 (–, –)	0.571	15 (1286)	− 188.9 (–, –)	0.604
< 5 mm	5 (445)	103.8 (–, –)	0.066	5 (445)	103.8 (–, –)	0.066	5 (445)	91.3 (–, –)	0.097	5 (445)	102.9 (–, –)	0.080
*Fractions*												
3	10 (779)	166.5 (146.2, 216.0)	0.008*	10 (779)	166.5 (146.2, 216.0)	0.008*	10 (779)	250.9 (231.6, 275.8)	< 0.0001*	10 (779)	207.3 (189.6, 232.7)	0.0002*
4	7 (662)	100.3 (–, –)	0.264	7 (662)	93.1 (87.8, 105.3)	**0.001**	6 (597)	97.4 (–, –)	0.283	6 (597)	106.3 (–, –)	0.140
5	4 (311)	122.6 (–, –)	0.243	4 (311)	113.9 (–, 1)	0.075	4 (311)	171.2 (–, –)	0.476	4 (311)	162.0 (–, –)	0.458
*Interval time*												
≤ 48 h	13 (1156)	64.7 (–, –)	0.604	12 (1091)	178.1 (–, –)	0.662	12 (1088)	22.4 (–, –)	0.605	11 (1023)	26.8 (–, –)	0.745
> 48 h	4 (290)	134.7 (124.6, 158.2)	**0.001**	4 (290)	127.0 (108.8, 167.1)	**0.001**	4 (290)	184.7 (156.5, 246.5)	**0.001**	4 (290)	166.0 (137.5, 240.2)	**0.002**
*Treatment equipment*												
LA	20 (1686)	− 624.4 (–, –)	0.948	19 (1621)	157.0 (–, –)	0.202	19 (1621)	336.3 (–, –)	0.440	18 (1556)	253.9 (–, –)	0.416
*Tissue heterogeneity correction*												
Yes	12 (998)	116.0 (106.8, 131.3)	**0.002**	12 (998)	102.5 (92.9, 115.3)	** < 0.0001**	12 (998)	142.4 (118.8, 177.0)	**0.001**	12 (998)	125.1 (105.9, 153.1)	**0.001**
No	4 (428)	148.3 (–, –)	0.063	3 (363)	131.4 (–, –)	0.053	4 (428)	195.8 (122.4, 368.5)	**0.020**	3 (363)	160.4 (–, –)	0.053
*Median follow-up time (months)*												
< 36 months	13 (1045)	120.8 (–, –)	0.367	12 (980)	103.3 (–, –)	0.741	13 (1045)	139.2 (–, –)	0.501	12 (980)	158.8 (–, –)	0.606
≥ 36 months	9 (816)	146.7 (-, –)	0.118	9 (816)	134.6 (102.2, 310.7)	**0.025**	8 (751)	206.8 (161.5, 342.2)	**0.006**	8 (751)	178.3 (137.9, 350.0)	**0.014**
			6			10			10			8

*BED* biologically effective dose, *s (p)* studies (patients), *ED90* the corresponding dose value when these parameters reached 90%, *CI* confidence interval, *VP* vacuum pillow, *AC* abdominal compressor, *LA* linear accelerator

Bold indicates significant dose effect relationship, *P* < 0.05

*Abnormal dose effect relationship, not account into cumulative number of significances

## Discussion

SBRT is an effective treatment strategy for early-stage NSCLC [31, 32]. Because of the volume effect of normal tissue tolerance and the small tumor volume in SBRT, the PTV of SBRT can be delivered a higher BED than conventional radiotherapy, and the BED range is relatively large. Therefore, it is necessary to establish the dose effect relationship between dose and local control rate, so as to obtain an optimal BED to guide clinical practice.

Through systematic review and meta regression analysis based on Probit model, this study found that there was no significant dose effect relationship between nominal BED_10_ or peripheral BED_10_ and 3 years local control rate, but there was a significant dose effect relationship between PTV central BED_10_ or PTV average BED_10_ and local control rate. This result is similar to that of Eriguchi et al. [5]. Through systematic review and meta-analysis, they determined that the correlation between central BED_10_ of PTV and tumor local control is stronger than that between peripheral BED_10_ and local control.

Although peripheral BED_10_ and nominal BED_10_ were significantly correlated with local control in subgroup analysis, the nominal BED_10_ or peripheral BED_10_ do not have the predictability of local control for whole included studies in our study. It seems to be in great contradiction with the previous radiobiological theory and clinical practice. However, after careful consideration, it is found that this result is reasonable. In conventional fractionated intensity modulated radiotherapy, the prescription dose is given with a unified normative standard, which encourages absorbed-dose homogeneity in the PTV. However, in SBRT, radiation oncologists seem to accept the extreme dose inhomogeneity within the PTV due to the characteristics of small field dosimetry and the advantage of dose inhomogeneity in PTV. In Radiation Therapy Oncology Group (RTOG) study 0813 and RTOG 0915, the prescription dose isodose line is selected at 60–90% of the maximum dose, that is, the maximum dose in the PTV is about 110–166% [23, 27, 33]. In terms of radiation physics, sufficiently high tumor dose inhomogeneity is also conducive to a sharp drop in the dose of normal tissues outside PTV [7]. At present, there is no uniform standard for the prescription dose method of SBRT. Some prescription doses are defined as isocenter dose [23, 24, 34], and some are defined as isodose lines surrounding 95% PTV volume [32, 35, 36]. At the same time, the degrees of heterogeneity of dose in PTV for different centers may be diverse. The maximum dose in PTV at some centers is about 120% of the prescribed dose [35], while some is more than 160% [31]. Therefore, for the same nominal dose, it may occur that the actual dose given to PTV is not comparable [37].

For the cohort study of the same prescription dose rule and PTV dose inhomogeneity, the correlation results between prescription dose BED_10_ or peripheral BED_10_ and tumor local or regional control have been obtained. Jeon et al. [38] found that PTV prescription dose BED_10_ > 100 Gy can obtain better 3 years regional control, 69.4% versus 100%, *P* = 0.004. Suzuki et al. [39] conducted a retrospective analysis on SBRT of 383 patients with early-stage NSCLC. The patients were divided into four groups based on GTV volume (11 cc) and PTV peripheral BED_10_ (85 Gy). The results demonstrated that among patients with GTV > 11 cc, the 5-years local control rate of patients with BED_10_ ≥ 85 Gy was better than that of patients with BED_10_ < 85 Gy, 65% versus 94%, *P* = 0.002.

For the central BED_10_, several studies have yielded correlations with local control. Onishi et al. [40] reviewed 245 patients from 13 Japanese institutions, and found that when BED_10_ < 100 Gy at the isocenter, the local recurrence rate was 26.4%, significantly higher than 8.1% in the group with BED_10_ ≥ 100 Gy, *P* < 0.05. For operable patients, the 3-years OS of patients with BED_10_ ≥ 100 Gy at isocenter was 88.4%, which was significantly higher than that of patients with < 100 Gy (69.4%, *P* < 0.05). Guckenberger et al. [41] performed a retrospective logistic regression analysis on 399 patients with NSCLC, and found that there was a significant dose effect relationship between PTV maximum BED_10_ and tumor local control: when PTV maximum BED_10_ reached 176 Gy, 90% of tumor control could be expected. This result is consistent with that of the Probit model in this study.

For the average BED_10_, several studies have suggested that there are significantly relationships between that and the local recurrence rate, progression free survival rate or OS. Kestin et al. [42] found that in the SBRT of NSCLC, the average dose BED_10_ of PTV was significantly correlated with the local recurrence rate. The 2-years local recurrence rate of patients with average BED_10_ < 125 Gy was 17%, while that of patients with average BED_10_ > 125 Gy was only 4%, *P* = 0.01. Zhao et al. [43] conducted univariate analysis on SBRT data of 1092 NSCLC patients, indicated that the average BED_10_ > 130 Gy was related to 97% local/lobar control, and suggested that it should be one of the aims of plan optimization. The univariate analysis results of Shamp et al. [44] showed that BED_10_ > 150 Gy in the center of the PTV was related to the improvement of disease-free survival (DFS) and OS, with *P* values of 0.014 and 0.0132 respectively. These results are similar to that of this study. This study reiterated the importance of the average BED_10_ of PTV. When the average BED_10_ dose reaches 140 Gy, a 3-years local control rate of 89.5% (86.7–91.0%) can be expected.

Based on the results of the Probit regression analysis in this study, combined with the definitions of central BED_10_ and average BED_10_, the ED90 of peripheral BED_10_ can be calculated is 123.9 Gy_BED10_. This result is slightly higher than that of Lee et al. [45]. Lee et al. tried to determine the optimal dose prescription of NSCLC SBRT through linear quadratic, universal survival curve, and regrowth models fitting. The results show that of the 3 models, the regrowth model provides the best fit to the clinical data. To achieve the maximal local control rate, the predicted physical dose schemes when prescribed at the periphery of the PTV are 43 ± 1 Gy in 3 fractions, 47 ± 1 Gy in 4 fractions, and 50 ± 1 Gy in 5 fractions. According to linear quadratic model calculation, the BED_10_ of the above dose schemes is 100.0–104.6 Gy. Our results pay more attention to the dose heterogeneity within the PTV, and suggest that a set of standard prescription dose specifications, including prescription dose definition and dose heterogeneity within the PTV, should be established.

From the results of our Probit model, they seem to give a false impression, that is, when the central BED_10_ or the average BED_10_ reaches 80 Gy or 70 Gy respectively, about 85% of the 3-years local control rate can be obtained. This is because, driven by previous dose effect conclusions [40], most BEDs are high, and data on lower BEDs is absence. In addition, there is also the possibility of publication bias, that is, it is difficult to publish the results with low local control rate at low BEDs or the authors are unwilling to publish.

This study has some limitations. There is a large heterogeneity in the patient cohort among the selected studies. These heterogeneities, including tumor location, size, number, and general status, are important confounding factors affecting local control of patients. In addition, the definition of local recurrence, the intervals and imaging modalities of follow-ups and recurrent diagnosis also affected the results. Due to the different ways of reporting in various literatures, the values of peripheral dose and central dose also have some uncertainties. Based on the Probit model in XLSTAT, it is not possible to conduct multifactor analysis for other factors besides the dose. The BED included in the Probit model analysis is calculated based on the LQ model. Therefore, the correctness and universality of the LQ model and the uncertainty brought by the value of α/β also affect the model results.

In conclusion, for NSCLC treated with SBRT, more attention should be paid to the central dose and average dose of PTV. The 3-years local control rate of 90.5% (87.5–92.1%) can be expected to be obtained at the central BED_10_ dose of 180 Gy; the 3-years local control rate of 89.5% (86.7–91.0%) can be expected to be obtained at the average BED_10_ dose of 140 Gy. A set of clear definition in the dose prescription should be established to ensure the effectiveness and comparability of treatment.

## Supplementary Information

**Additional file1**. **Table S1**: Search strategies of databases. **Figure S1**: PRISMA Flow diagram of the included studies.

## Data Availability

All data, models, or code generated or used during the study are available from the corresponding author by request.

## References

[1] Ceniceros L, Aristu J, Castañón E (2016). Stereotactic body radiotherapy (SBRT) for the treatment of inoperable stage I non-small cell lung cancer patients. Clin Transl Oncol.

[2] Videtic GM, Stephans KL (2010). The role of stereotactic body radiotherapy in the management of non-small cell lung cancer: an emerging standard for the medically inoperable patient?. Curr Oncol Rep.

[3] Hamaji M (2020). Surgery and stereotactic body radiotherapy for early-stage non-small cell lung cancer: prospective clinical trials of the past, the present, and the future. Gen Thorac Cardiovasc Surg.

[4] Chang JY, Mehran RJ, Feng L (2021). Stereotactic ablative radiotherapy for operable stage I non-small-cell lung cancer (revised STARS): long-term results of a single-arm, prospective trial with prespecified comparison to surgery. Lancet Oncol.

[5] Eriguchi T, Takeda A, Nemoto T (2022). Relationship between dose prescription methods and local control rate in stereotactic body radiotherapy for early stage non-small-cell lung cancer: systematic review and meta-analysis. Cancers.

[6] Klement R, Sonke J, Allgäuer M (2020). Correlating dose variables with local tumor control in stereotactic body radiation therapy for early-stage non-small cell lung cancer: a modeling study on 1500 individual treatments. Int J Radiat Oncol Biol Phys.

[7] Hong L, Garg M, Lasala P (2011). Experience of micromultileaf collimator linear accelerator based single fraction stereotactic radiosurgery: tumor dose inhomogeneity, conformity, and dose fall off. Med Phys.

[8] Tateishi Y, Takeda A, Horita N (2021). Stereotactic body radiation therapy with a high maximum dose improves local control, cancer-specific death, and overall survival in peripheral early-stage non-small cell lung cancer. Int J Radiat Oncol Biol Phys.

[9] Baumann P, Nyman J, Hoyer M (2009). Outcome in a prospective phase II trial of medically inoperable stage I non-small-cell lung cancer patients treated with stereotactic body radiotherapy. J Clin Oncol.

[10] Fakiris AJ, McGarry RC, Yiannoutsos CT (2009). Stereotactic body radiation therapy for early-stage non-small-cell lung carcinoma: four-year results of a prospective phase II study. Int J Radiat Oncol Biol Phys.

[11] Takeda A, Sanuki N, Kunieda E (2009). Stereotactic body radiotherapy for primary lung cancer at a dose of 50 Gy total in five fractions to the periphery of the planning target volume calculated using a superposition algorithm. Int J Radiat Oncol Biol Phys.

[12] Baba F, Shibamoto Y, Ogino H (2010). Clinical outcomes of stereotactic body radiotherapy for stage I non-small cell lung cancer using different doses depending on tumor size. Radiat Oncol.

[13] Ricardi U, Filippi AR, Guarneri A (2010). Stereotactic body radiation therapy for early stage non-small cell lung cancer: results of a prospective trial. Lung Cancer.

[14] Timmerman R, Paulus R, Galvin J (2010). Stereotactic body radiation therapy for inoperable early stage lung cancer. JAMA.

[15] Shirata Y, Jingu K, Koto M (2012). Prognostic factors for local control of stage I non-small cell lung cancer in stereotactic radiotherapy: a retrospective analysis. Radiat Oncol.

[16] Rosen LR, Fischer-Valuck BW, Katz SR (2014). Helical image-guided stereotactic body radiotherapy (SBRT) for the treatment of early-stage lung cancer: a single-institution experience at the Willis-Knighton Cancer Center. Tumori.

[17] Lindberg K, Nyman J, Riesenfeld Kallskog V (2015). Long-term results of a prospective phase II trial of medically inoperable stage I NSCLC treated with SBRT: the Nordic experience. Acta Oncol.

[18] Nagata Y, Hiraoka M, Shibata T (2015). Prospective trial of stereotactic body radiation therapy for both operable and inoperable T1N0M0 non-small cell lung cancer: Japan clinical oncology group study JCOG0403. Int J Radiat Oncol Biol Phys.

[19] Navarro-Martin A, Aso S, Cacicedo J (2016). Phase II trial of SBRT for stage I NSCLC: survival, local control, and lung function at 36 months. J Thorac Oncol.

[20] Shaverdian N, Tenn S, Veruttipong D (2016). The significance of PTV dose coverage on cancer control outcomes in early stage non-small cell lung cancer patients treated with highly ablative stereotactic body radiation therapy. Br J Radiol.

[21] Tsurugai Y, Kozuka T, Ishizuka N (2016). Relationship between the consolidation to maximum tumor diameter ratio and outcomes following stereotactic body radiotherapy for stage I non-small-cell lung cancer. Lung Cancer.

[22] Sun B, Brooks ED, Komaki RU (2017). 7-year follow-up after stereotactic ablative radiotherapy for patients with stage I non-small cell lung cancer: results of a phase 2 clinical trial. Cancer.

[23] Cummings MA, Ma SJ, Hermann G (2018). Comparison of single- and five-fraction regimens of stereotactic body radiation therapy for peripheral early-stage non-small-cell lung cancer: a two-institution propensity-matched analysis. Clin Lung Cancer.

[24] Karasawa K, Hayakawa S, Machitori Y (2018). Accelerated hypofractionated radiotherapy versus stereotactic body radiotherapy for the treatment of stage I nonsmall cell lung cancer-a single institution experience with long-term follow-up. Technol Cancer Res Treat.

[25] Ma SJ, Cummings M, Serra LM (2018). Three- versus five-fraction regimens of stereotactic body radiotherapy for peripheral early-stage non-small-cell lung cancer: a two-institution propensity score-matched analysis. Clin Lung Cancer.

[26] Raghavan G, Shaverdian N, Chan S (2018). Comparing outcomes of patients with early-stage non-small-cell lung cancer treated with stereotactic body radiotherapy based on frailty status. Clin Lung Cancer.

[27] Bezjak A, Paulus R, Gaspar LE (2019). Safety and efficacy of a five-fraction stereotactic body radiotherapy schedule for centrally located non-small-cell lung cancer: NRG oncology/RTOG 0813 trial. J Clin Oncol.

[28] Nicosia L, Reverberi C, Agolli L (2019). Long term results of single high dose stereotactic body radiotherapy in the treatment of primary lung tumors. Sci Rep.

[29] Devpura S, Feldman AM, Rusu SD (2021). an analysis of clinical toxic effects and quality of life as a function of radiation dose and volume after lung stereotactic body radiation therapy. Adv Radiat Oncol.

[30] Ryuno Y, Abe T, Iino M (2022). High-dose stereotactic body radiotherapy using CyberKnife® for stage I peripheral lung cancer: a single-center retrospective study. Radiat Oncol.

[31] Tsurugai Y, Takeda A, Sanuki N (2019). Stereotactic body radiotherapy for patients with non-small-cell lung cancer using RapidArc delivery and a steep dose gradient: prescription of 60% isodose line of maximum dose fitting to the planning target volume. J Radiat Res.

[32] Nittala MR, Duggar WN, Mundra E (2021). Single institution experience of stereotactic body radiation therapy in non-small cell lung cancer: comparison of two dose regimes and a perspective on ideal dose regimens. Cureus.

[33] Grills IS, Mangona VS, Welsh R (2010). Outcomes after stereotactic lung radiotherapy or wedge resection for stage I non-small-cell lung cancer. J Clin Oncol.

[34] Watanabe K, Katsui K, Sugiyama S (2021). Lung stereotactic body radiation therapy for elderly patients aged ≥ 80 years with pathologically proven early-stage non-small cell lung cancer: a retrospective cohort study. Radiat Oncol.

[35] Zhang R, Guo Y, Yan Y (2021). A propensity-matched analysis of survival of clinically diagnosed early-stage lung cancer and biopsy-proven early-stage non-small cell lung cancer following stereotactic ablative radiotherapy. Front Oncol.

[36] Steber CR, Hughes RT, Soike MH (2021). Stereotactic body radiotherapy for synchronous early stage non-small cell lung cancer. Acta Oncol.

[37] Merlotti A, Bonomo P, Ragona R (2021). Dose prescription in SBRT for early-stage non-small cell lung cancer: are we all speaking the same language?. Tumori.

[38] Jeon W, Ahn SJ, Kim YC (2018). Correlation of biologically effective dose and the tumor control in Stage I (<5 cm) non-small cell lung cancer with stereotactic ablative radiotherapy: a single institutional cohort study. Jpn J Clin Oncol.

[39] Suzuki O, Mitsuyoshi T, Miyazaki M (2014). Dose-volume-response analysis in stereotactic radiotherapy for early lung cancer. Radiother Oncol.

[40] Onishi H, Araki T, Shirato H (2004). Stereotactic hypofractionated high-dose irradiation for stage I nonsmall cell lung carcinoma: clinical outcomes in 245 subjects in a Japanese multiinstitutional study. Cancer.

[41] Guckenberger M, Klement RJ, Allgäuer M (2016). Local tumor control probability modeling of primary and secondary lung tumors in stereotactic body radiotherapy. Radiother Oncol.

[42] Kestin L, Grills I, Guckenberger M (2014). Dose-response relationship with clinical outcome for lung stereotactic body radiotherapy (SBRT) delivered via online image guidance. Radiother Oncol.

[43] Zhao L, Zhou S, Balter P (2016). Planning target volume d95 and mean dose should be considered for optimal local control for stereotactic ablative radiation therapy. Int J Radiat Oncol Biol Phys.

[44] Shamp SJ, Sheikh S, Chang T (2021). Stereotactic body radiotherapy (SBRT) for T2N0 (>3 cm) non-small cell lung cancer: outcomes and failure patterns. J Radiosurg SBRT.

[45] Lee P, Loo BW, Biswas T (2021). Local control after stereotactic body radiation therapy for stage i non-small cell lung cancer. Int J Radiat Oncol Biol Phys.

