# Supplementary material for: Dose–effect relationship of stereotactic body radiotherapy in non-small cell lung cancer patients
Source: Radiat Oncol. 2022 Dec 23;17:211. doi: 10.1186/s13014-022-02183-3 (PMC9789627; doi:10.1186/s13014-022-02183-3)
Supplement: Supplementary file 1 — Additional file1. Table S1: Search strategies of databases. Figure S1: PRISMA Flow diagram of the included studies. [file 13014_2022_2183_MOESM1_ESM.docx]

| Databases | Search strategies | Search outcomes |
| --- | --- | --- |
| The PubMed Database | #1 Search: stereotactic ablative radiotherapy[Title/Abstract]  #2 Search: stereotactic body radiotherapy[Title/Abstract]  #3 Search: stereotactic radiotherapy[Title/Abstract]  #4 Search: stereotactic body radiation therapy[Title/Abstract]  #5 Search: SBRT[Title/Abstract]  #6 Search: SABR[Title/Abstract]  #7 Search: #1 OR #2 OR #3 OR #4 OR #5 OR #6  #8 Search: non-small cell lung cancer[Title/Abstract]  #9 Search: nonsmall cell lung cancer [Title/Abstract]  #10 Search: NSCLC[Title/Abstract]  #11 Search: #8 OR #9 OR #10  #12 #7 AND #11 Filter: Language: English | 872  3,393  3,275  3,601  5,698  1,225  11,075  71,466  2,751  55,871  80,985  2,001  1,926 |
| Web of Science Core Collection | #1 TI=(stereotactic ablative radiotherapy)  #2 TI=(stereotactic body radiotherapy)  #3 TI=(stereotactic radiotherapy)  #4 TI=(stereotactic body radiation therapy)  #5 TI=(SBRT)  #6 TI=(SABR)  #7 #1 OR #2 OR #3 OR #4 OR #5 OR #6 #8 TI=(non-small cell lung cancer) #9 TI=(nonsmall cell lung cancer) #10 TI=(NSCLC) #11 #8 OR #9 OR #10 #12 #7 AND #11 Filter: Language: English | 614  2,087  4,480  2,131  1,200  285  7,525  46,278  1,843  7,222  53,330  998  962 |
| The Cochrane Library | #1 (stereotactic ablative radiotherapy): ti, ab, kw  #2 (stereotactic body radiotherapy): ti, ab, kw  #3 (stereotactic radiotherapy): ti, ab, kw  #4 (stereotactic body radiation therapy): ti, ab, kw  #5 (SBRT): ti, ab, kw  #6 (SABR): ti, ab, kw  #7 #1 OR #2 OR #3 OR #4 OR #5 OR #6  #8 (non-small cell lung cancer): ti, ab, kw  #9 (nonsmall cell lung cancer): ti, ab, kw  #10 (NSCLC): ti, ab, kw  #11 #8 OR #9 OR #10  #12 #7 AND #11 | 313  782  1,412  785  802  261  1750  14,261  9,718  10,500  15,252  398 |

Table S1

Search strategies of databases


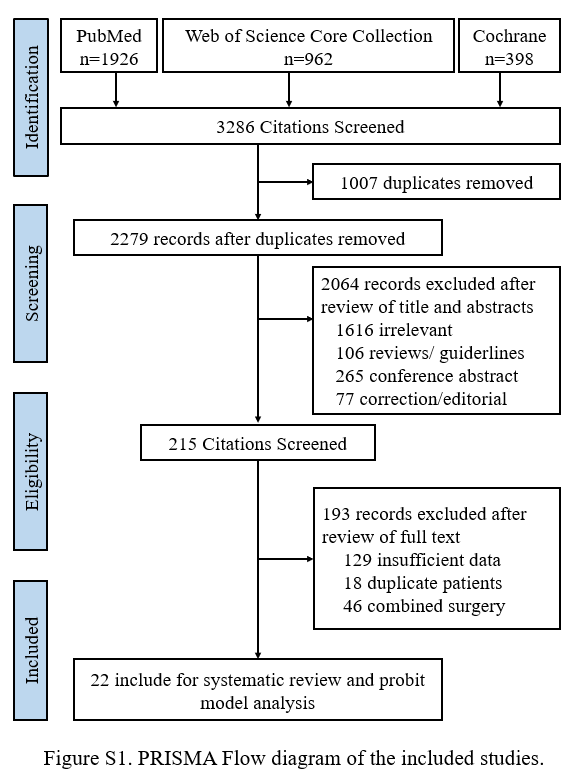


**Fig. S1.** PRISMA Flow diagram of the included studies.
